# Rubber in Prosthetic Dentistry

**Published:** 1888-03

**Authors:** Robert R. Russell

**Affiliations:** Nashvile, Tenn.


### Rubber in Prosthetic Dentistry.

BY ROBERT R. RUSSELL, NASHVILE, TENN.

Mr. President:—It seems, that whether among the works of nature, or of man, there can be no benefit without its corresponding injury, no blessing without a curse, no invention or discovery of use, which in some degree, is not also of detriment to mankind.

When the genius of Goodyear gave the world the method of utilizing the juices of the Syphona Cahucha, he conferred a very great benefit. India rubber has been adapted to so many uses that it seems wonderful how we ever got along without it, and impossible to discover a substitute for it.

From the garment, impervious to moisture, to the band that drives myriad-wheeled machinery; from the flint-hard handle of the surgeon's knife, to the elastic bulb that equalizes the spray of the atomizer; in almost every department of science, art, or ordinary life, this invention has added to the comfort and convenience of mankind. A restless ingenuity has applied rubber not only to these external uses, but has not been content until it has put it into our mouths. The ease with which it is adapted to the uses of dentistry, has caused every other consideration to be over-looked, neglected, or forgotton. The idea of melting a substance into a mould, hardening it by vulcanization into exact fit and shape, was hailed as a new era in dentistry, a boon to the mechanical worker and to the patient. The former was freed from the necessity of acquiring any knowledge of metallurgy; of any skill to alloy, to solder, to roll, to swage, or to mold metals. He no more looked into the fiery eye of the crucible filled with liquid gold to be run into ingots and rolled into shining plates.

The beautiful art which mounted the polished plate with its coronal of dental pearls, and placed it like an ornate jewel into the human mouth, that gate of health and portal of speech, was forgotten or despised.

The dentist was turned into a maker of tiles, whose sole art is to put rubber into a sort of kiln and bake it. Into the gate, opened by this invention, rushed an indiscriminate and uneducated herd, fully equal to the task of cooking India-rubber. All that was required for their equipment was a cooking machine, called a vulcanizer. Such results, in the mechanism of artificial dentures, were achieved, as to cause every dentist to hang his head in shame, and every patient literally to gnash his teeth in agony. What the effect has been upon honorable dentistry goes without saying. What the result has been upon the people who depend upon the profession for relief from the evils and inconveniencies of endentulous mouths, it would take a volume to tell. Not only is it true that the mouths of patients have been injured, but in many instances their general health has been impaired. What is it, that has been put into the mouths of the people? Who can tell? The manufacturers of dental rubber refuse to make known their recipes. The Dentist was then required by the necessities of the case, to place for daily use into his patients mouths a substance, the composition of which he could only have an imperfect knowledge; there, to be acted on by the fluids and absorbents of the human mouth; to react, by its chemical composition upon them, and exert some unknown influence over all the delicate structures of nerves and blood vessels; and to contribute some unknown compound to the salivation and mastication of food on the way to the stomach; to make blood for the nutrition and sustenance of human life. Into the mouths of the robust and the delicate, male and female, old and young, without any consideration apparently, but that it fit the cavity and held the teeth in place, this vulcanite stranger was recklessly pushed.

But enough is now known of the composition of dental rubber, and of the local and systemic effects upon those who wear it, to put its advocates upon the defensive.

The crude caoutchouc after being macerated, washed, and dissolved, is hardened by the addition of sulphur and colored with vermillion, the white oxide of zinc, or ivory black, as is required to obtain the color and shade desired. The more sulphur the harder the product. For the red rubber the coloring matter is the sulphuret of mercury, (vermillion) and the pink rubber is made by decreasing the amount of the sulphuret of mercury and adding the white oxide of zinc. The published formulæ of Dr. Wildman show that red rubber consists of

| | | |
|---|---|---|
| Caoutchouc | 48 | parts |
| Sulphur | 24 | " |
| Sulphuret of mercury | 36 | " |

The pink rubber of

| | | |
|---|---|---|
| Caoutchouc | 48 | parts |
| Sulphur | 24 | " |
| White oxide of zinc | 30 | " |
| Sulphuret of mercury | 10 | " |

Maroon rubber contains 28 per cent. of vermillion, although fraudulently sold as free from it.

Sulphur acts physiologically, as a laxative, *diaphoretic* and resolvent, and when taken into the system by external or internal application for any considerable time, in quantities however minute, its presence can be detected by the *exhalations* of the skin and lungs, and in the feces, urine, and milk.

Coming in contact with the soda of the bile, it is rendered soluble and is carried into the circulation by the fatty matters of the alimentary canal, which hold it in solution and sometimes produces extreme depression of the nervous and circulatory systems. None of the properties of sulphur are lost in being mixed with caoutchouc and the sulphuret of mercury. Nor does vulcanizing affect it, except to harden it into a compact mass.

The effect of the sulphuret of mercury upon the system is the same as pure mercury, except in degree; its composition being in the proportion of 40 ounces Troy of mercury to 8 ounces of sulphur. Mercury penetrates all the tissues and being non-nutritive, hinders their formation, causes their waste, or hastens their destruction. It diminishes the proportion of the fibrin and the red corpuscles of the blood, produces morbid sensibility to cold, cutaneous eruptions, eczema, intractable ulcers, particularly in the mouth and throat; salivation, fetid breath, mental and nervous depressions. Its continuous exhibition in any quantity deranges the functions, impairs nutrition, and enfeebles the health. It remains in the tissues, and may be extracted from the secretions during life and from the blood after death. It is nocuous even when breathed in an atmosphere impregnated by the fumes from the furnaces where it is smelted, and the proportion of abortions, still-births and *chachetic* children is excessive in localities where the air is contaminated by emanations from the molten metal. These are the well known and direful effects of mercurial poisoning where the mineral acts by its own force, and they are produced whether it is taken by the mouth, or by cutaneous absorption. It seems probable that when united with sulphur as a vehicle, it would not be less apt to work its malign effects, when applied constantly by mercurial inunction to the mucous membrane of the mouth ; conveyed into the system by absorption and inhalation, and incorporated in the process of salivation and mastication with the food conveyed into the stomach.

That the effects thus naturally and scientifically to be expected from the use of sulphur and vermillion in the dentures of the last twenty years have followed, the experience of every competent and candid dentist will abundantly attest.

How often have you seen as the result of the use of a rubber plate, the red line of salivation along the gingival attachments of the teeth, tumefactions of the lips and fauces, spongy condition of the gums, sodden tongue with the impress of the teeth upon the edges, ulcers attacking the cheeks and throat, intractable, or in healing adhering to the adjacent parts; absorption of the alveolar process and bony substance of the jaw, irritation and inflammation of the mucous membrane, gastric irritation and mysterious derangements of the general health, and sometimes alarming functional disorders, particularly in cases of delicate and highly organized women resulting in direful uterine woes.

And how often have you seen all these conditions bettered, or cured, by discarding the vulcanite plate? The dentist has freduently relieved where the physician failed, and the reason was that the physician overlooked, or was ignorant of the condition of his patients mouth, whether from diseased teeth, or gums, or poisonous plates, and gave remedies in vain.

The fact is, and it is one under valued by the professions, both of medicine and dentistry, that the condition of the health often depends to a greater degree upon the condition of the mouth, than upon any other single condition whatsoever.

The injurious effects of vulcanite has been an open secret in the profession for years. It has been published in Dental Journals, spoken in Dental Associations, and still it is used more extensively than any other material. The only way, if there is a way, for the dentist to acquit himself from blame in its use, is to warn his patient, that although it is cheap in price, it is dear property which generally has to be paid for in suffering and ill health.

I have gone into almost an elementary treatise upon the known properties and medicinal effects of the component parts of vulcanite for the double purpose of educating both the people and the profession. For if the profession knows the facts, as it is presumed to do, it hides its knowledge under a bushel. And as "faith without works is dead," so also is knowledge that is never applied. The abnormal irritation resulting in almost every instance of the first use of a rubber plate, and indeed in the continued use of it, was thought by some long ago, and I regret to say by some who use it now, to be attributed to the rough surface of the plate applied to the mucous membrane. But that this is preposterous ignorance, or mendacity, is proven by the fact that if the plate is polished the irritation is increased. It is undoubtedly true that the fact that vulcanite is almost a perfect non-conductor of heat and cold, should long since have concluded the argument against it use, and shut the doors of conscientious dentistry against it. In this category is also celluloid.

The capillaries, not covered by the vulcanized plate, are subject to the contraction of cold and the expansion of heat, while those covered by the non-conductor are not thus affected by either heat or cold. This causes the blood in these capillaries to collect and expand into lagoons ; and thence come all the evils of unequal circulation, irritation, congestion, ulceration, and absorption. This plate cooks the tissues, obtunds the sensory and paralyzes the vaso motor nerves, destroys the process, leaving in its place a flabby flexible ridge with a flat hard palate, which will not retain or support any denture in its place ; hence the mouth is rendered irremediably edentulous, unless indeed the victim is willing to go through life with a slip-shod horror in his mouth, continually slipping down to view to be excruciatingly sucked up into place to the discomfort of the wearer and nausea of the spectator. Nor is this the full indictment of filth. Vulcanite in cooling from 320 degrees contracts more than any metal heated to that point, shrinks from the porcelain, and leaves a cavity in the rear of each tooth. This, with use, becomes filled with rotting food debris that pollutes the saliva with its oozings and mingles its carrion-juices with the food; the mouth is made a stench-trap, and the stomach a cess-pool, reeking with odors vile.

It is due to humanity, to science, to the dental profession, that it should take a stand against the use of substances that are deleterious to health, not to mention the consequent degradation of its professional tone and standard of ethics by the introduction of modes whose recommendation is their cheapness, and opening the doors to charlatans and botchers who get poor prices for poor work, and who deride and decry any attempt to rescue dentistry and dental patients from the injurious effects on reputation and purse, person and health, by discarding unfit and poisonous material.

With all such it is a fight for existence. To discard rubber would unfrock them. Othelo's occupation would be gone. If the inventive genius of dentists renders it possible to dispense with plates in partial dentures by bridge-work, they rush into print as reviewers to assure the public that the majority of enlightened and progressive dentists are against such questionable operations. The triumphant and indisputable fact is, that those substances should alone be used for plates that while they answer all purposes of the mechanical construction and the effective use of artificial teeth, are not destructive of the mouth or deleterious to the health of those who wear them.

Porcelain is such a substance. Gold, the king of metals, whether in a monetary, manufacturing, or scientific sense, is to be preferred to all metals. Platinum is excellent, and aluminium also. The latter, as a cheap material, is within the reach of all.

This metal was only a laboratory product till 1858, when it was rendered capable of being utilized and manufactured in quantity. First evolved as the metalic base of alumina, then of cryolite, and lastly of mica, feldspar, slate, and clay. Dana says, "Nearly all the rocks except limestones and many sandstones are literally ore-beds of aluminium." It is the most abundant metal in the earth's crust, and seems destined to become the most useful of all. It is four times lighter than silver, and nine times lighter than gold; it is a good conductor of heat and electricity; does not oxidize; has no action on water at ordinary temperature; will not tarnish or blacken in sulphuretted hydrogen. Of all the acids hydrochloric alone will dissolve it. Nitric acid refuses to attack it. It also is soluable in caustic potash or soda. It alloys with most all metals. It is readily rolled into plates. It can be melted and poured into molds like any fusible alloy, and when thus used it holds the teeth as firmly as by any process, thus obviating the necessity of using vulcanite for that purpose.

Science hails aluminium as the coming metal, and predicts the day when it will supercede iron itself,—the foundation of man's civilization,—and startles the world with the assertion that we are on the threshold of the day when that mineral will be as utterly supplanted and lost as the stone of the aborigenes. Aluminium gives blue to the sapphire, green to the emerald, yellow to the topaz, red to the ruby, brown to emery, and gray, blue, and black to the slates and clays. It will be the most abundant and cheapest of all the metals. But, gentlemen, the main fight is, the fight for honorable dentistry, is to put the ban on the "cheap and nasty," and if we can do no more, convince the world that in our profession

Cleanliness is nae pride,
Dirt is nae honesty.

---

Where ice cannot be procured, water may be cooled by wrapping the pitcher containing it in a towel of loose texture which has been previously impregnated with ammonium nitrate (*and dried*), and moistening this with water. The same towel may be used repeatedly, being dried thoroughly beforehand each time.—*Phar. Era.*

